# Dr. Arthur's System of Separating the Teeth as Reviewed by O. A. Jarvis, D. D. S., of New York

**Published:** 1872-10

**Authors:** J. B. Hodgkin

**Affiliations:** VA.


AMERICAN JOURNAL

OF

DENTAL SCIENCE.

Vol. VI. THIRD SERIES—OCTOBER, 1872. No. 6.

### ARTICLE I.

### *Dr. Arthur's System of Separating the Teeth as Reviewed by O. A. Jarvis, D. D. S., of New York.*

BY J. B. HODGKIN, D. D. S., OF VA.

The monograph of Dr. Arthur has called forth a sharpness of contradiction, and a severity of denunciation from its opponents, which has the appearance of the effort of men who fear that their occupation will be gone by its adoption, rather than the criticism of those who are seeking the truth; and I am sure no one will hail with greater pleasure than Dr. A. these efforts at answering him, as such strictures serve to spread before a wider circle of readers his doctrines. The effort at frowning down this little book is only a repetition of history. Professional ostracism has ever awaited the man who discovers truth, and so much the more if that truth be unpalatable, and to the selfish in a guise calculated to diminish his profits by its adoption.

These strictures of Dr. Jarvis as published in the *Dental Cosmos*, are sweeping. I trust they may not be found unanswerable. He says: "It is not well to be in haste to add text, standard, or popular works on dentistry, until principles are better understood, and practice better defined"

and he " questions the wisdom of advocating with so much boldness a practice so much at variance with nature, and receiving such doubtful support from results in practice. ' As Dr. Jarvis toward the end of his article proposes a new plan of separating of his own invention, as a substitute for the method under discussion, it seems to me that here he is not quite consistent, and if the " principles" on which he practices are " not well defined ," surely he should hesitate before criticising the methods of others. He should not find fault with " a practice at variance with nature" until it is established that the practice he advocates is in harmony with nature. Indeed the whole practice of dentistry, so far as it relates to the subject of filling teeth and inserting artificial ones, is as much at variance with nature as anything proposed by Dr. Arthur. If it is natural to fill a tooth at all, if any naturalness is found in any part of the process, whether by " soft foil," " contour fillings," restoring the shape of the tooth," " following the type of nature," or anything about it, from the inception of decay to the completion of the filling, then I must plead ignorance.

Dr. Jarvis' first point is that " separation answers the desired and only in so far as it renders the (proximal) surfaces self-cleansing." If he means by this, that we are to dispense with the tooth-brush as a result of this system, I can hardly think him in earnest; if he means that the act of mastication is thus converted into a tooth cleansing process, then he does not understand Dr. Arthur's method.

His objection is: "1st, It is unnatural." As previously stated, decay is unnatural, filling teeth is artificial, and no part of the process has any pretentions to being called a natural process.

If our critic will reproduce dentine and enamel in a carious tooth, I will acknowledge *that* a natural process; but, to say that teeth should not be separated for superficial decay because it is unnatural, is simply absurd. As well say that stumps of teeth should not be extracted preparatory to the insertion of artificial teeth, because it is unnatural.

As to the statement that it "does not harmonize with the plan of the original Artist," and that "progressive dentists are increasingly inclined to follow the teachings and preserve the types nature gives," it is sufficient to say that until a substance can be found that will so adhere to the tooth, so as to become practically a part of it, "restoring the type," is premature as a rule. Few gold fillings have a sufficiently tenacious hold in a molar cavity to withstand the force of mastication, if the type of the tooth is restored.

Dr. Jarvis continues:

2nd. "The point of contact in the normal condition of the teeth, is almost invariably single, or generally more limited in extent than can be secured by artificial separation;" and this he thinks should not be removed, as no subsequent point of contact can be made so small. As Dr. Arthur does not propose separation until this point of contact shows unmistakable signs of decay, (Dr. Jarvis himself will I think admit that softening of the enamel is decay,) and as he does not propose to leave the surfaces in contact, there can be no "subsequent point of contact." (See page 98 of Dr. Arthur's book.)

Our critic thinks this removal of the point of contact of the proximal surfaces of the teeth, "an irretrievable injury, for which there can be no possible compensation." Does he think that an irretrievable misfortune, for which there can be no compensation—the condition of the teeth of those of his patients whose dentures are by nature more or less separated, and would he go to work with regulating apparatus to bring such teeth in juxtaposition?

My own incisors and canines—the only perfect teeth I have—are so scattered naturally. I fear had I fallen in childhood into Dr. J's hands, I should have had these so regulated as to be decayed and filled also.

Dr. Jarvis will say "but that is their normal condition; I do not meddle with nature."

According to his theory the "type" should be followed, and this type is almost uniformly, "teeth in contact;" if we are to follow the type, and avoid artificial separation, then it is logical and philosophic to follow the type and bring together when separated naturally.

Would it not be well for those who harp so much on this subject of "types" to consider that it is a question of expediency alone with which we have to deal; to remember that the question is not what we would like to accomplish for the sake of present appearances, that should be our highest aim, but what is most expedient for the permanent welfare of our patient.

"3rd. The injury to the gums and process by the pressure of food in the process of mastication, and the violent pressure of the tooth in one direction and the next instant in the opposite direction; the present pain and the resulting periodontitis and probable absorption, the annoyance of the tongue by the artificial angles."

I revert to the cases just spoken of—those dentures naturally separated. Not only do I write from personal experience, my own incisors being naturally so separated, but I have, in common I suppose with every dentist, met with numbers of persons whose teeth, like mine, are separated naturally. I have yet to hear them speak of any annoyance from that source, and I certainly experience none myself. I find nothing of the driving of teeth violently, first one way and the next moment the other way, to their permanent injury. On the contrary I find this separation a source of comfort and convenience, and am well satisfied that to this separation alone is due the exemption of these teeth from decay. It is a frequent remark with me on seeing such teeth, "You are fortunate;" and the reply is often, "Yes, I can keep them so nice and clean; I wish they were all so." But in no case have I heard of this condition of the teeth being any annoyance, or resulting in any injury to the "gums or process," and I certainly think there is as good an opportunity in these cases for the "violent pressure of the teeth in one direction, and the next instant in the opposite direction," as when the teeth are separated artificially. And indeed as the teeth are unsupported on the buccal or lingual surfaces by opposing teeth, why are they not driven in those directions by the lateral motion of the jaws, and loosened, as Dr. J. insists they will be, in the other direction?

"The annoyance to the tongue" is scarcely worth noticing, especially as our author says on the next page that "we have not yet to learn that people will get used to a mouthful of broken and decayed teeth." How much more readily will they become accustomed to the slight alteration proposed?

Dr. Jarvis says, "If we are going to appeal to the people for their verdict, we have it already in the universal horror and undying dread with which they have regarded this practice for the past thirty years."

This is a sweeping statement and deserves some consid eration. Is it the truth? I am aware that to grasp a huge, badly cut file, and seizing the jaw with one hand, proceed to saw away at a sensitive, badly decayed molar, is an operation, which as practised "thirty years" ago, and to this day by some, (perhaps by Dr. Jarvis,) is an operation calculated to inspire dread. But is there not a better way?

But may not the popular prejudice, (Dr. Jarvis calls it "testimony,") on the injurious effects resulting from "breaking the enamel," arise from some erroneous notions on the subject?

Will Dr. J. inform us on what speciality the popular mind is sufficiently well informed to be able to pass judgment? On what speciality is not the popular mind crowded with ludicrous errors? If among physicians there are many to whom the science of medicine is a mass of ill-digested crudities, how much more full of ignorance is the "popular" mind? How many can our author find among his unprofessional friends, who can give a reason for their faith in this or that remedy?

Once for all let it be understood that the scientific man never courts or submits to an appeal to the popular verdict.

It is impossible for them to be judges, and Dr. J. ought to know enough of history to know this.

I am not certain but that the popular idea of the injurious effects of the " mutilation" had its origin in the errroneous teachings of dentists of former days; dentists who, " wise in their own conceit," could not see anything out of their own practice. And has Dr. J. yet to learn how long an error, once fairly started, may run before its falsity is made apparent? " The teeth generally do, and may be expected to shift their position, unless prevented by antagonists."

The " antagonists" of teeth are those which oppose them in the other jaw. Teeth in juxtaposition are not antagonists. But I think that teeth isolated by the extraction of contiguous fellows much more liable to this shifting than teeth separated by Dr. Arthur's method. Where a tooth moves forward, it is caused as a rule by the removal of its anterior fellow. If the roots of teeth are sound and intact they act as a restraining force in preventing forward movement; and I think the " forward slope" of the molars so frequently seen, will be found nearly always to be caused by the loss of the first permanent molars or a bicuspid. This slope, once given by the loss of these teeth, will of course crowd them forward.

Dr. J. says: 5th. " It is unnecessary; the care on the part of the dentist and the patient, which Prof. A. requires, (p. 159,) if timely applied, would probably secure the desired results, and there is a better plan of separation * * * by enlarging the inner or second V, leaving the grinding surfaces intact."

Dr. Jarvis proposes this as a substitute for the method of Dr. Arthur and he says he practises it. If this be true what becomes of the type he was " increasingly" along with the " more progressive dentists," " inclined to follow?" He says indeed that this plan would give abundant opportunity for inspection, but he says nothing about its availability in keeping these surfaces clean—a fatal objection, if it had no other. But it may be that the patients of Dr. J.

have different teeth from those that fall into my poor hands, for I find it exceedingly difficult as a rule to force the thinest wedge or tape tightly between molars or bicuspids for the purpose of gaining space, and if this be so how can "the same care required by Dr. Arthur," be taken with them? That care is, as he quotes Dr. A., (pp. 157–59,) to use tape "several times a day," and "carefully examine the surfaces at short intervals." Such cleansing is manifestly impossible with teeth as close together as molars and bicuspids generally are; and generally impracticable with incisors also.

He continues; "Let us try again the filling plan pronounced by Dr. Arthur a failure, as applied to the proximate surfaces of the molars and bicuspids. Imperfect operations are a more prolific source of failure than all others combined."

I readily grant this, and in no style of filling is it as difficult to avoid imperfection as in restoring the partly lost crowns of molar and bicuspid teeth. I leave this to the conscience of every dentist in the land. I do not believe it possible for the majority of practicing dentists, to make as a rule, perfect "contour fillings;" it requires an amount of skill not possessed by more than one, perhaps in ten, and not perhaps attainable by them. Dr. J. may, perhaps say that they should retire from the profession, and give place to better men; but can the better men be found?

We all know that we may with moderate talent, and in a fair amount of time make a satisfactory filling in the proximal surface of a tooth, if that surface is plane and freely open to inspection. But the difficulty of the case is very much increased, when an effort is made to build up and restore the shape of a molar tooth, with the cavity perhaps under the edge of the gum, with much of the strength of the tooth gone, and with the surface of the wall, as it is frequently found, concave on its vertical face—depressed in the centre of the crevical wall, high at its sides—if such a tooth is built out so as to restore its original shape, and follow the "type," the certainty of perfectly finishing the crevical wall is doubtful. The operator is reduced to rely on the sense of touch, and his work is mere guess-work; for if he has restored the shape of the tooth, it will be impossible for him to see very well his work at this point on account of the bulging crown; and the probabilities are that his costly structure will prove in a year or so a total failure.

I do not wish to be understood as waging a war against contour fillings, I leave that for abler hands, or perhaps for another time, but I cannot let the opportunity pass of calling attention to one more fact that weakens this style of work in my regard very much. No molar tooth is firm in its socket, but has a perceptible movement. No gold is rigid, no matter how densely packed or welded; it will bend if strain sufficient is put on it. If a tooth is built out, the gold occupying the dovetailed space which is orthodox, and resting against the adjacent tooth, the movement of the tooth in mastication gives an opportunity for the gold to bend and draw out of the cuts, often necessarily slight, into which it is built, and this it is apt to do if great force is put on it, or if it does not bend, the slight walls may and sometimes do split, and a total ruin occur to a filling which, but for the fatal "building out" might have lasted for many years.

I pass by Dr. J's "clerical friend," as he is not a dentist, and not qualified to enter the discussion; and pass even the statement that cutting away the inscisors result in deformity, with the remark that if Dr. J. does not know how to separate inscisors after the plan of Dr. Arthur, so that it will result in much less deformity than building out such a tooth, if he will come to see me I will show him some samples.

"How," Dr. Jarvis proceeds, "are we to determine when decay is irretrievable? How often do we see cases where the destructive influences have been removed and the frail character no longer appears."

Decay of the tooth is irretrievable when softening of the enamel has occured. Softening itself is decay. I think Dr. J. will concede this much, and if he has seen as a rule, or even occasionally, softening of the enamel arrested, or not followed by decomposition of the dentine, *when no operation had been performed to arrest it*, then I can only say that his patients have peculiar teeth.

That Dr. Arthur's "plan of artificial separation of the teeth as proposed in his book, is not necessary; is not the best treatment; is a failure;" is a series of statements Dr. J. has made, but, as I think, utterly failed to substantiate. That it is a perfect success I do not say; no human patchwork of nature is. Man is mortal, and frailty and decay is stamped on all his works. Exposed as the teeth are to decaying agencies, and valuable as we know them to be, it is wonderful that such progress has been made in the way of their preservation. If Dr. Arthur's system results in any improvements on our present system—if anything in it will aid in developing the large-hearted dentist we all hope to see everywhere, we should hail his little book as light on our upward path to such perfection as we may hope to attain. Dr. Arthur is certainly fond of his pet,—we all are of our special creations,—and if he be deemed by some to have swung his pendulum past the centre in the onward sweep of Advance, let us see to it that we are not so far behind as some seem to think he is too far ahead in new ideas.

---

